# Identification of molecular targets for esophageal carcinoma diagnosis using miRNA-seq and RNA-seq data from The Cancer Genome Atlas: a study of 187 cases

**DOI:** 10.18632/oncotarget.16051

**Published:** 2017-03-09

**Authors:** Jiang-Hui Zeng, Dan-Dan Xiong, Yu-Yan Pang, Yu Zhang, Rui-Xue Tang, Dian-Zhong Luo, Gang Chen

**Affiliations:** ^1^ Department of Pathology, First Affiliated Hospital of Guangxi Medical University, Nanning, Guangxi Zhuang Autonomous Region 530021, China

**Keywords:** esophageal carcinoma, DEMs, DEGs, TCGA, diagnosis

## Abstract

Esophageal carcinoma (ESCA) is one of the most common malignancies worldwide, and its pathogenesis is complex. In this study, we identified differentially expressed miRNAs (DEMs) and genes (DEGs) of ESCA from The Cancer Genome Atlas (TCGA) database. The diagnostic values of DEMs were determined by receiver operating characteristic (ROC) analyses and validated based on data from Gene Expression Omnibus (GEO). The top five DEMs with the best diagnostic values were selected, and their potential targets were predicted by various in silico methods. These target genes were then identified among the DEGs from TCGA. Furthermore, the overlapping genes were subjected to protein-protein interaction (PPI) analysis, Gene Ontology (GO) and Kyoto Encyclopedia of Genes and Genomes (KEGG) pathway analyses. The miRNA-transcription factor (TF) regulatory relations were determined using CircuitsDB and TransmiR. Finally, the regulatory networks of miRNA-TF and miRNA-gene were constructed and analyzed. A total of 136 DEMs and 3541 DEGs were identified in ESCA. The top five DEMs with the highest area under the receiver operating characteristic curve (AUC) values were miRNA-93 (0.953), miRNA-21 (0.928), miRNA-4746 (0.915), miRNA-196a-1 (0.906) and miRNA-196a-2 (0.906). The combined AUC of these five DEMs was 0.985. The KEGG analysis with 349 overlapping genes showed that the calcium signaling pathway and the neuroactive ligand-receptor interaction were the most relevant pathways. The regulatory networks of miRNA-TF and miRNA-gene, including 38 miRNA-TF and 560 miRNA-gene pairs, were successfully established. Our findings may provide new insights into the molecular mechanisms of ESCA pathogenesis. Future research will aim to explore the role of novel miRNAs in the pathogenesis and improve the early diagnosis of ESCA.

## INTRODUCTION

Esophageal carcinoma (ESCA) is one of the most common malignancies worldwide. ESCA is the seventh leading cause of cancer-correlated mortality in men in the United States in 2016 [1]. It accounts for 4% of all cancer deaths in men [1]. The five-year survival rate of patients with ESCA is currently 20%, but 38% of cases are diagnosed at a late stage, for which the five-year relative survival rate is 4% [1]. The high mortality rate was associated with the late diagnosis and poor treatment response [2, 3]. Early diagnosis and treatment can decrease the mortality and increase the five-year relative survival. Therefore, it is important to identify novel biomarkers for the early diagnosis of ESCA and develop new targeted therapies.

Large quantities of miRNAs have been observed expressing differentially in cancer tissues, which indicate prospective diagnostic values. Some studies showed a number of miRNAs (miR-21, miR-143, miR-145 [4] and miR-92b [5, 6]) as potential biomarkers for the diagnosis of ESCA. However, the clinical value of miRNAs in ESCA remains largely unraveled.

The Cancer Genome Atlas (TCGA) provides a new source of information to identify novel biomarkers. TCGA is a community resource project, and its data have been widely used in cancer research. The TCGA dataset contains 2.5 petabytes of data describing tumor and matched normal tissues from 33 types of cancer with more than 11000 patients. The TCGA ESCA data were updated on May 16, 2016. The number of miRNA and mRNA expression values increased from 1064 to 1881 and 20531 to 60483, respectively. There have been only two studies thus far that used TCGA data regarding ESCA to investigate the expression profiles of miRNAs and genes. Zhan et al. [7] found that 45 miRNAs and 2962 genes were differentially expressed in ESCA compared with normal esophageal tissues, which was based on the TCGA data from July 2014. Additionally, Zhao et al. [8] only focused on the differentially expressed miRNAs (DEMs) in ESCA and investigated their prognostic value, based on the TCGA data from June 2015. No study has explored the diagnostic value of DEMs in ESCA based upon the TCGA data. Therefore, it is possible and urgent to identify sensitive and specific biomarkers to identify potential pathogenic mechanisms and improve the accuracy of early diagnosis of ESCA based on TCGA data.

This study screened for the presence of DEMs and differentially expressed genes (DEGs) in ESCA. We also performed a receiver operating characteristic (ROC) analysis to investigate the diagnostic value of DEMs in ESCA and validated the top five DEMs based on data from Gene Expression Omnibus (GEO). The overlapping genes, which were largely representative of the potential target genes of the DEMs, were assessed using protein-protein interaction (PPI) analysis, Gene Ontology (GO) and Kyoto Encyclopedia of Genes and Genomes (KEGG) pathway analysis. The regulatory networks of miRNA-transcription factor (TF) and miRNA-gene were successfully established. Our study could provide a meaningful contribution to exploring the mechanisms of esophageal carcinoma pathogenesis and defining new biomarkers for early diagnosis and treatment.

## RESULTS

### DEMs and DEGs in ESCA based on TCGA data

**Figure 1:**
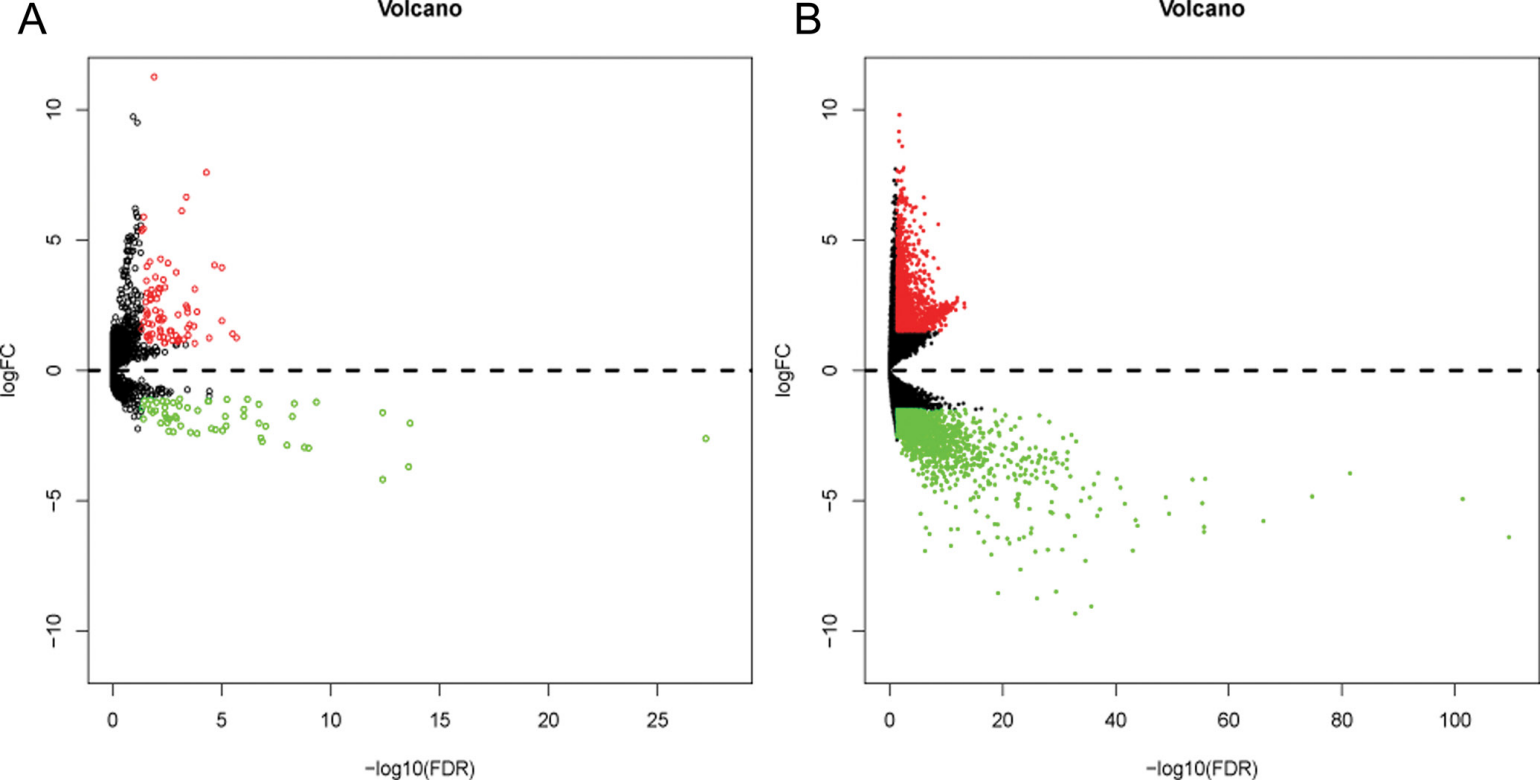
Volcano plot of differentially expressed miRNAs (DEMs) and differentially expressed genes (DEGs) in esophageal carcinoma (ESCA) and normal esophageal samples (**A**) Volcano plot of differentially expressed miRNAs (DEMs). Volcano plot was generated using the gplots package in Bioconductor. DEMs with log_2_FoldChange (log_2_FC) > 1 were labeled in red; DEMs with log_2_FoldChange (log_2_FC) < -1 were in green (*P* < 0.05). (**B**) Volcano plot of differentially expressed genes (DEGs). DEGs with log_2_FoldChange (log_2_FC) > 1.5 were shown in red; DEGs with log_2_FoldChange (log_2_FC) < -1.5 were in green (*P* < 0.05).

**Figure 2:**
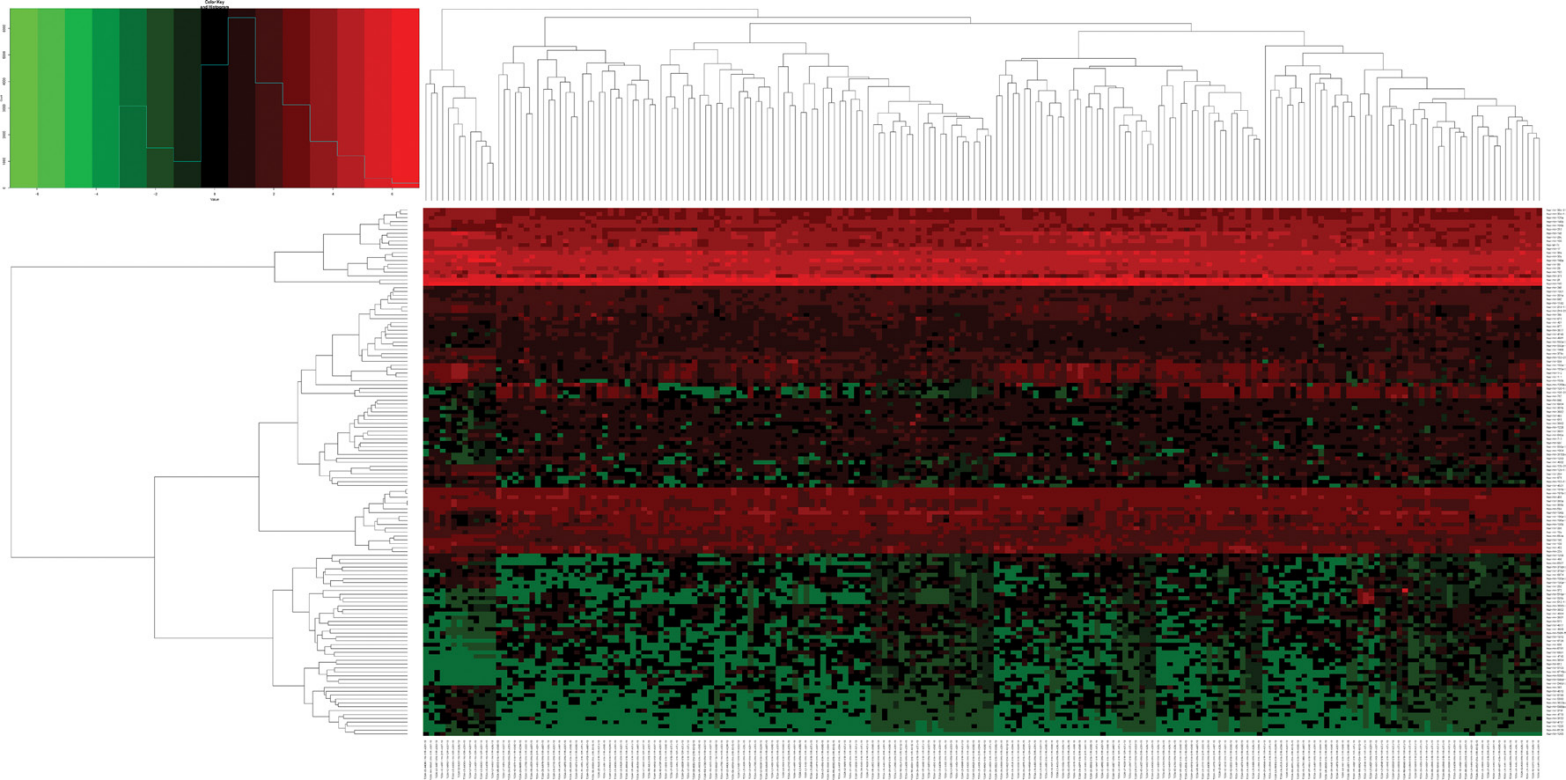
Heatmap of differentially expressed miRNAs (DEMs) in esophageal carcinoma (ESCA) and normal esophageal samples Heatmap was generated using the gplots package in Bioconductor. DEMs with log_2_FoldChange (log_2_FC) > 1 were labeled in red; DEMs with log_2_FoldChange (log_2_FC) < -1 were in green (*P* < 0.05).

**Figure 3:**
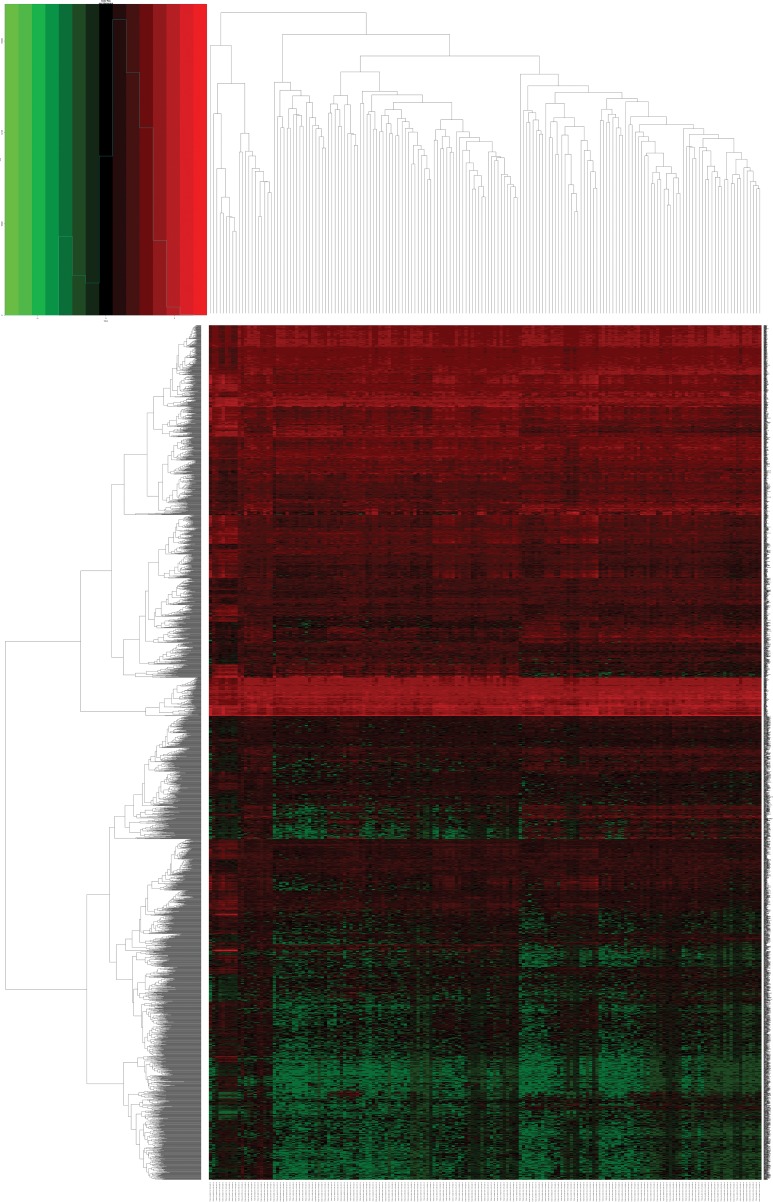
Heatmap of differentially expressed genes (DEGs) in esophageal carcinoma (ESCA) and normal esophageal samples Heatmap was drawn using the gplots package in Bioconductor. DEGs with log_2_FoldChange (log_2_FC) > 1.5 were shown in red; DEMs with log_2_FoldChange (log_2_FC) < -1.5 were in green (*P* < 0.05).

Altogether, 136 miRNAs were considered as DEMs in ESCA based upon a *p*-value < 0.05, FDR < 0.05 and |log_2_FoldChange| > 1 using the edgeR package in Bioconductor, including 79 up-regulated and 57 down-regulated miRNAs (Supplementary Table 1, Figures 1 and 2). In total, 3541 genes were identified as DEGs in ESCA according to similar criteria except for |log_2_FoldChange| > 1.5, including 1688 up-regulated and 1853 down-regulated genes (Figures 1 and 3).

### The diagnostic value of DEMs

**Figure 4:**
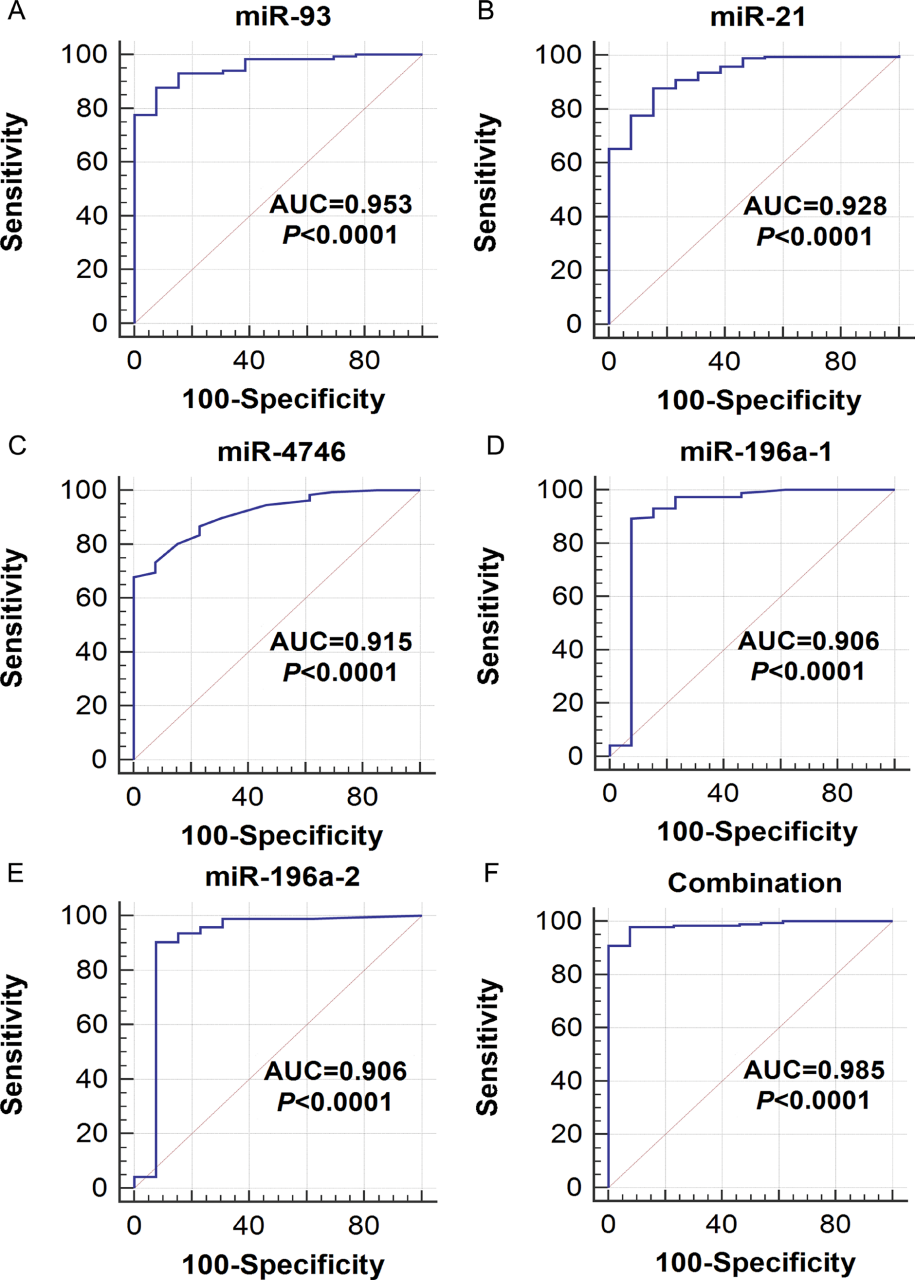
The receiver operating characteristic (ROC) curves of the top five differentially expressed miRNAs (DEMs) in esophageal carcinoma (ESCA) ROC curves were drawn using MedCalc software. AUC: area under the ROC curve. (**A**) miR-93, (**B**) miR-21, (**C**) miR-4746, (**D**) miR-196a-1, (**E**) miR-196a-2, (**F**) Combination of the five DEMs.

**Figure 5:**
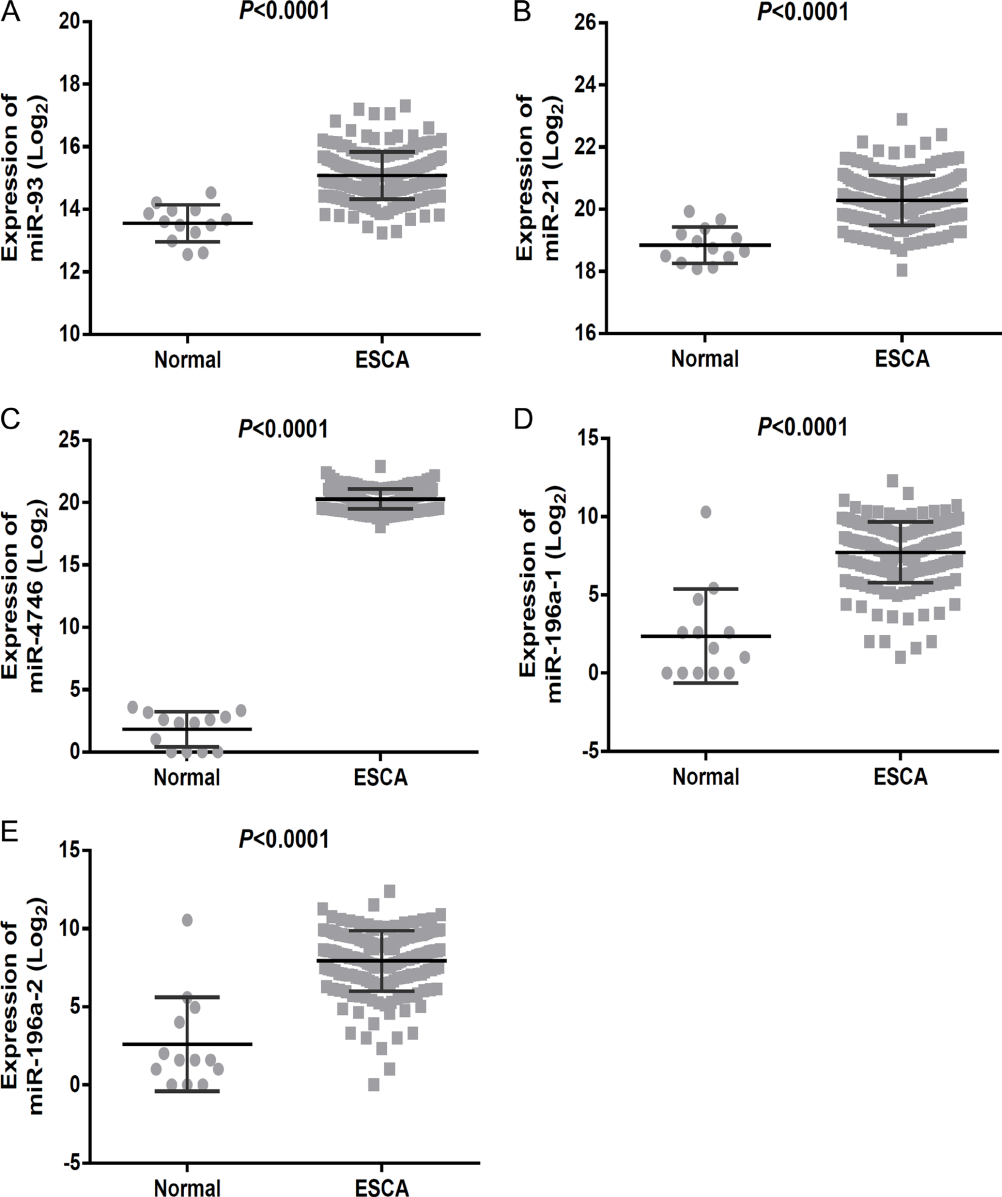
Expression of the top five differentially expressed miRNAs (DEMs) in esophageal carcinoma (ESCA) The scatter diagrams showed the expression level of DEMs in the 187 cases of ESCA and 13 normal esophageal samples. The scatter diagrams were drawn using the GraphPad Prism software. (**A**) miR-93, (**B**) miR-21, (**C**) miR-4746, (**D**) miR-196a-1, (**E**) miR-196a-2.

The ROC analysis of the DEMs was performed (Supplementary Table 1). The top five areas under the ROC curves (AUCs) for DEMs were 0.953 (miR-93), 0.928 (miR-21), 0.915 (miR-4746), 0.906 (miR-196a-1) and 0.906 (miR-196a-2) (Table 1, Figure 4). We also combined these five DEMs to evaluate the potential diagnostic value for ESCA, and the pooled AUC reached 0.985, which provided a higher diagnostic efficiency compared to individual DEMs. We next examined the expression level of the five DEMs in non-cancerous esophageal and ESCA tissues using GraphPad Prism (version 6.01, Figure 5). The top five DEMs, including five up-regulated miRNAs (miR-93, miR-21, miR-4746, miR-196a-1 and miR-196a-2), were selected for further analysis.

**Table 1:** The top five differentially expressed miRNAs (DEMs) in esophageal carcinoma (ESCA)

miRNA ID	LogFC*	*p*-value	FDR**	AUC***
**miR-93**	1.394654953	4.44E-08	3.18E-06	0.953
**miR-21**	1.246193842	2.81E-08	2.12E-06	0.928
**miR-4746**	1.896011225	1.72E-07	9.94E-06	0.915
**miR-196a-1**	1.993005619	0.000557766	0.007777738	0.906
**miR-196a-2**	1.943227278	0.000604741	0.008131613	0.906

*FC: fold change

**FDR: fault detection rate

***AUC: area under the ROC curve

### Validation of the top five DEMs

**Figure 6:**
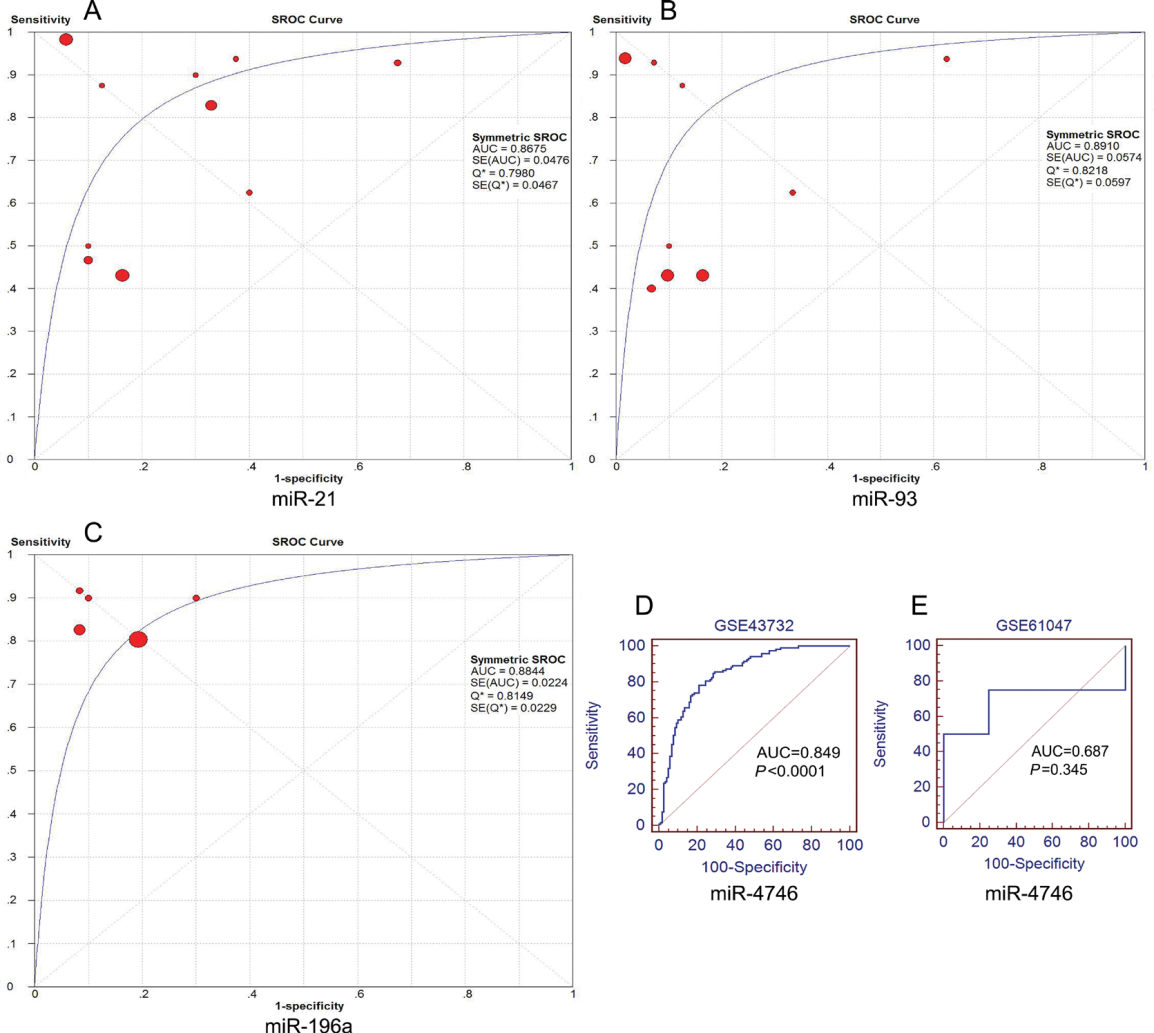
Summary receiver operating characteristic (SROC) plots of miR-21, miR-93 and miR-196a and the receiver operating characteristic (ROC) curves of miR-4746 SROC curves were drawn using Meta-DISc software. ROC curves were drawn using MedCalc software. (**A**) miR-21, (**B**) miR-93, (**C**) miR-196a, (**D**) miR-4746 (GSE43732), (**E**) miR-4746 (GSE61047).

**Figure 7:**
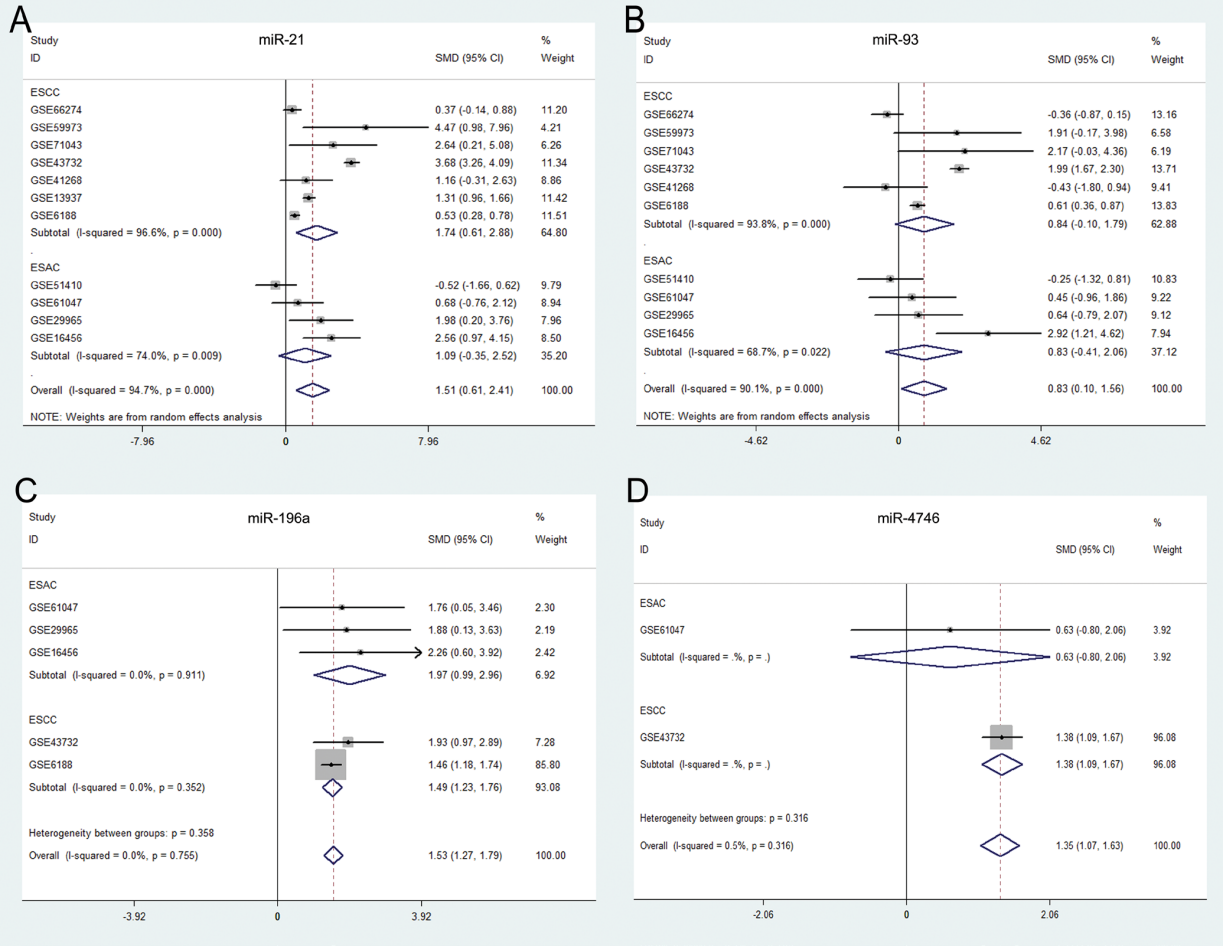
Forest plots of the top five differentially expressed miRNAs (DEMs) in esophageal carcinoma (ESCA) Forest plots were drawn using STATA software (version 12.0). (**A**) miR-21, (**B**) miR-93, (**C**) miR-196a, (**D**) miR-4746.

A total of 11 miRNA microarray datasets were included in the present study. The areas under summary receiver operating characteristic (sROC) curves were 0.868 (miR-21), 0.891 (miR-93) and 0.884 (miR-196a). Since data from only two microarrays were available for miR-4746, we could perform the meta-analysis. The original AUCs of miR-4746 were 0.849 (GSE43732) and 0.687 (GSE61047), respectively (Figure 6). Our results suggested that the expression levels of miR-21, miR-93, miR-196a and miR-4746 were remarkably higher in ESCA specimens than those in normal controls (miR-21: standardized mean difference (SMD) = 1.51, 95% CI: 0.6~2.4; *P* = 0.001; miR-93: SMD = 0.83, 95% CI: 0.1~1.56; *P* = 0.025; miR-196a: SMD = 1.53, 95% CI:1.27~1.79; *P* < 0.0001; miR-4746: SMD = 1.35, 95% CI: 1.07~1.63; *P* < 0.0001), which were consistent with our previous results on the basis of TCGA (Table 3, Figure 7).

**Table 2:** Relevant transcription factors (TFs) regulated by eight differentially expressed miRNAs (DEMs)

miRNA ID	TransmiR	CircuitsDB
**miR-93**	E2F1,MYC	NF-Y, TEF-1, EGR, SRF
**miR-21**	SMAD3, IL1B, TGFB1, AP-1,BMP6, ERS1, Gfi1, NFIB, PTEN, REST, STAT3,AKT, Foxo3a, RAS/ERK, BMPR1a, BMPR1b, EGFR, NFKB1, DDX5, TCF7L2, ETV5, REL, RELA	TEF-1
**miR-4746**	/	/
**miR-196a-1**	/	MYB, HSF2, GATA, STAT5A, ER, T3R,RORALPHA2
**miR-196a-2**	HMGA1	/

**Table 3:** The standard mean deviation (SMD) of the top five differentially expressed miRNAs (DEMs) in ESCA

Group	Cancer type	Number of patients	SMD*	SMD (95%CI)		*P* value	Heterogeneity	
				Lower	Upper		*I^2^*(%)	*P* value
**miR-21**	Overall	11	1.51	0.61	2.40	0.001	94.7	< 0.0001
	ESCC**	7	1.74	0.61	2.88	0.003	96.6	< 0.0001
	ESCA***	4	1.09	−0.35	2.52	0.138	74	0.009
**miR-93**	Overall	10	0.83	0.10	1.56	0.025	90.1	< 0.0001
	ESCC	6	0.84	−0.10	1.79	0.08	93.8	< 0.0001
	ESCA	4	0.83	−0.41	2.06	0.189	68.7	0.022
**miR-196a**	Overall	5	1.53	1.27	1.79	< 0.0001	0	0.755
	ESCC	2	1.49	1.23	1.76	< 0.0001	0	0.352
	ESCA	3	1.97	0.99	2.96	< 0.0001	0	0.911
**miR-4746**	Overall	2	1.35	1.07	1.63	< 0.0001	0.5	0.316
	ESCC	1	1.49	1.23	1.76	< 0.0001	/	/
	ESCA	1	0.63	−0.80	2.06	0.389	/	/

*SMD: standard mean deviation

**ESCC: esophageal squamous cell carcinoma

***ESCA: esophageal carcinoma

### Overlapping genes from DEGs and predicted targets

**Figure 8:**
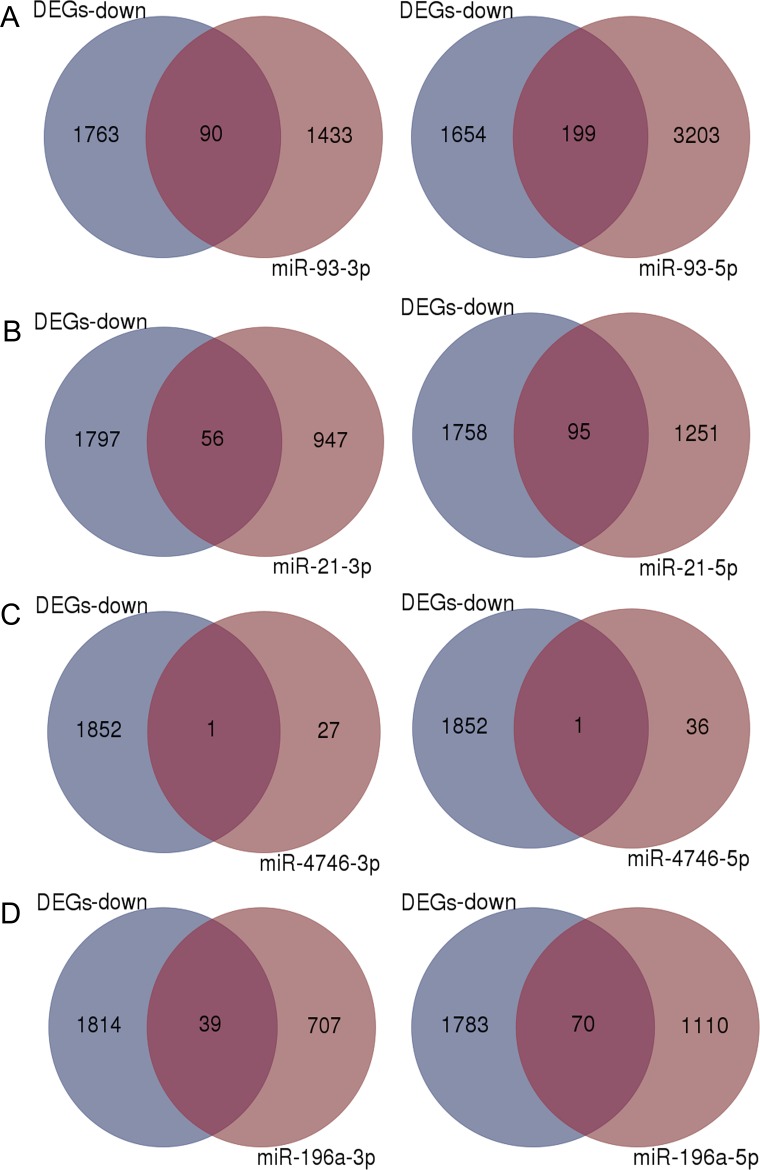
Venn diagrams of overlapping genes from differentially expressed genes (DEGs) and predicted target genes Blue: Down-regulated DEGs, Light purple: predicted target genes of each miRNA, Dark purple: overlapping genes. (**A**) miR-93, (**B**) miR-21, (**C**) miR-4746, (**D**) miR-196a.

Different numbers of predicted target genes were obtained for each DEM (Figure 8), and the total number of the target genes was 6115. The intersections between the target genes of the four up-regulated miRNAs (miR-93, miR-21, miR-4746 and miR-196a) and 1853 down-regulated DEGs are shown in Figure 8A–8D and Supplementary Table 2. We also examined the combination of all overlapping genes and 349 genes were identified.

### PPI network construction

**Figure 9:**
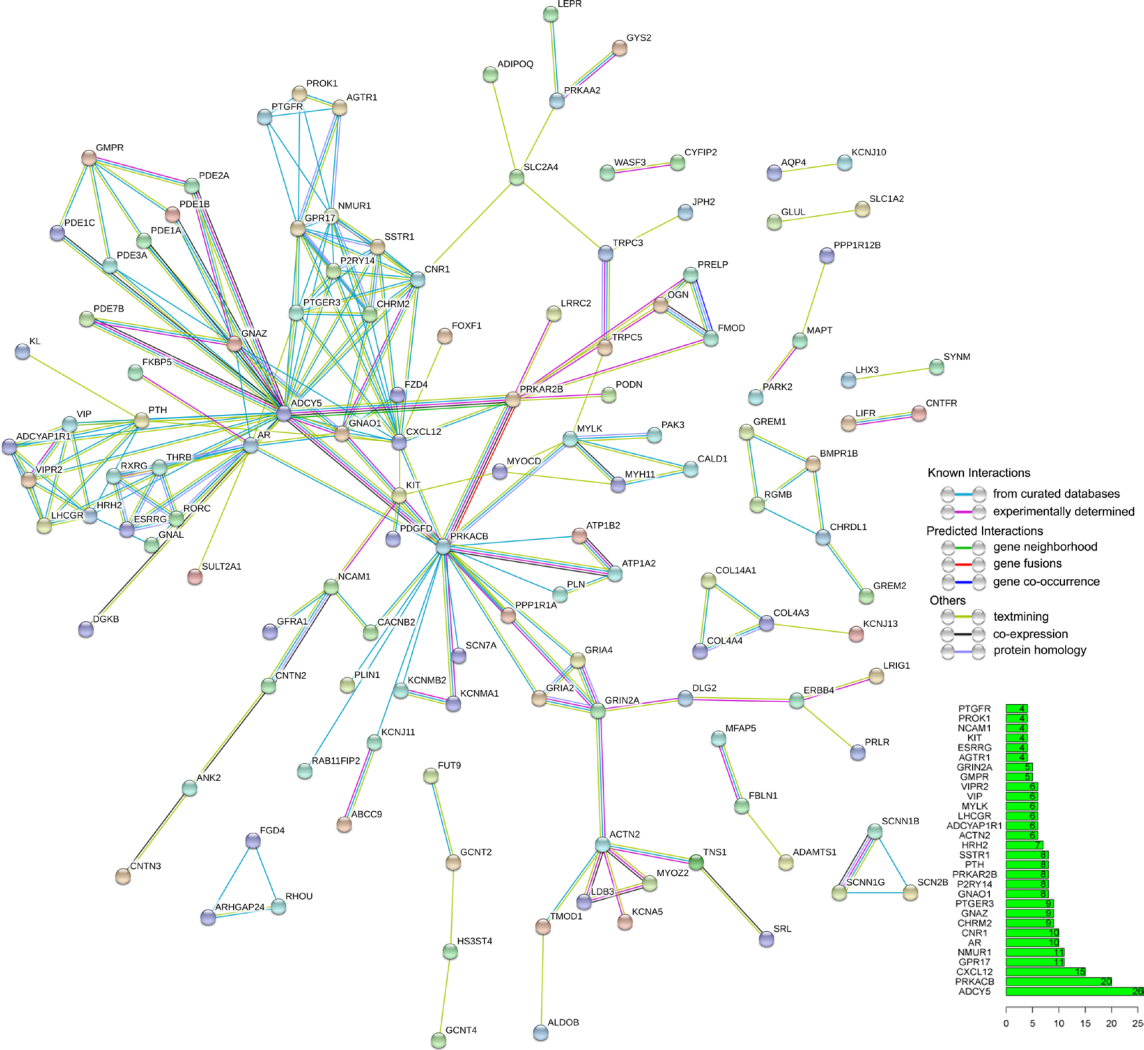
Protein-protein interaction (PPI) network of overlapping genes PPI network was drawn using STRING online tool. The minimum required interaction score was 0.7 (high confidence). Disconnected nodes were hidden in the network. The bar graph showed the number of connected nodes for the top 30 genes.

PPI network was constructed with nodes representing the proteins and edges depicting associated interactions. PPI network was established with 349 overlapping genes containing 349 nodes and 213 edges (Figure 9).

### Validation of the top five DEMs target genes

**Figure 10:**
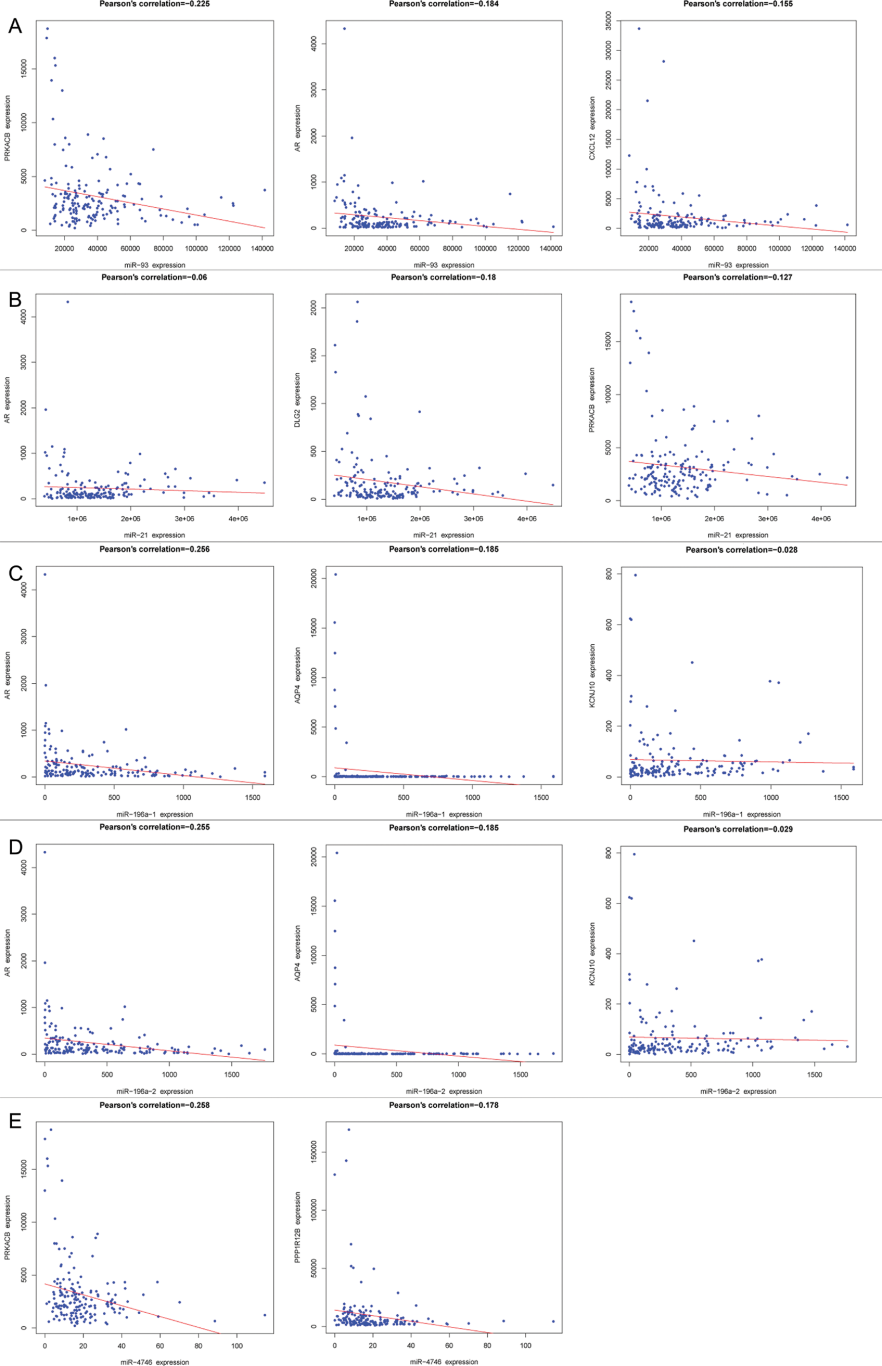
The correlation between the top three hub genes and each differentially expressed miRNA (DEM) Pearson's correlation plots were drawn using R language. (**A**) miR-93 (PRKACB, AR and CXCL12), (**B**) miR-21(AR, DLG2 and PRKACB), (**C**) miR-196a-1 (AR, AQP4 and KCNJ10), (**D**) miR-196a-2 (AR, AQP4 and KCNJ10), (**E**) miR-4746 (PRKACB and PPP1R12B).

PPI network was performed to the overlapping genes of each DEM (miR-93, miR-21, miR-196a-1 and miR-196a-2). The correlation between the top five hub genes and each DEM was negative and the top three were shown in Figure 10. The negative correlations between protein kinase cAMP-activated catalytic subunit beta (PRKACB), protein phosphatase 1 regulatory subunit 12B (PPP1R12B) and miR4746 were evidently presented in Figure 10 too.

### GO and KEGG pathways of overlapping genes

**Figure 11:**
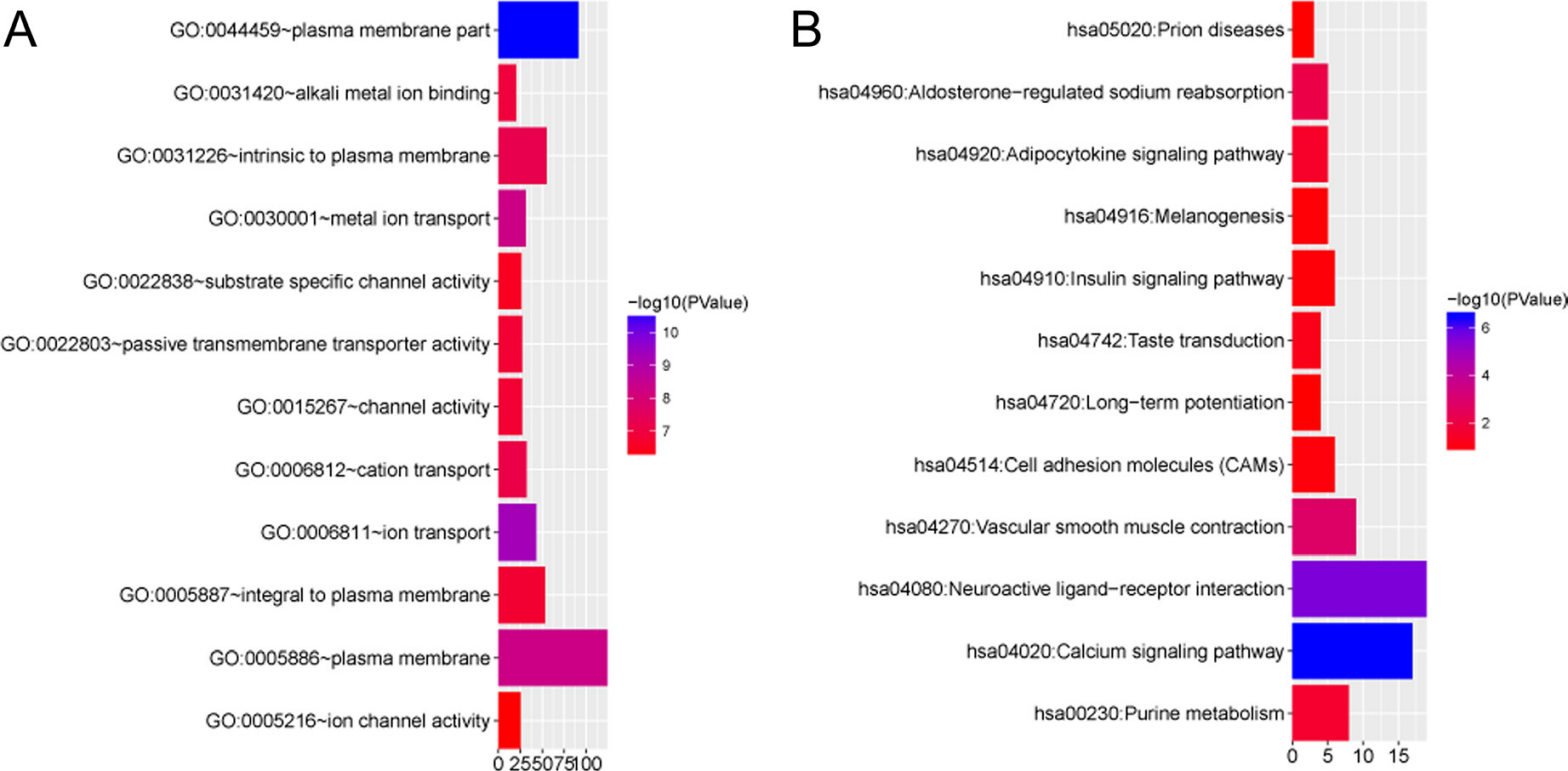
Gene Ontology (GO) annotation and Kyoto Encyclopedia of Genes and Genomes (KEGG) pathways of overlapping genes GO Annotation and KEGG Pathways were generated using the ggplot2 package in R language. The horizontal axis represented the number of genes. Column color represented the -log_10_(*P*-Value). Blue: high degree of enrichment, red: low degree of enrichment. (**A**) GO annotation of overlapping genes, (**B**) KEGG pathways of overlapping genes.

To gain insight into the biological roles of the 349 overlapping genes encoding the four DEMs in ESCA, we performed GO annotation and KEGG pathway analyses. The results of GO annotation suggested that the main functions of these overlapping genes were related to the plasma membrane, while the results of KEGG analysis indicated that the overlapping genes were mainly correlated with the calcium signaling pathway and the neuroactive ligand-receptor interaction (Figure 11).

### The regulatory networks of miRNA-transcription factor (TF) and miRNA-gene

**Figure 12:**
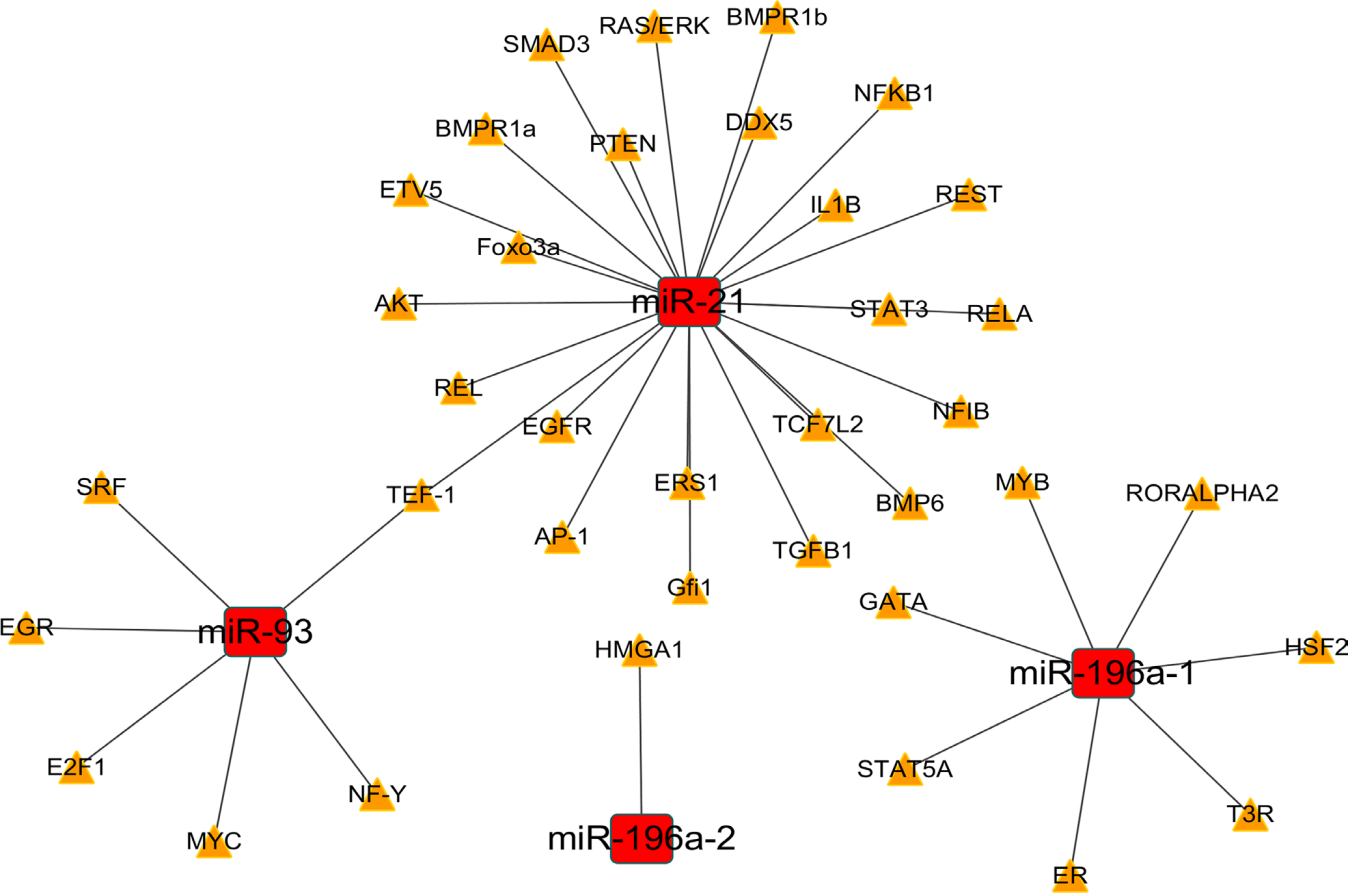
The regulatory network of miRNA-(transcription factor) TF The regulatory network of miRNA-TF in esophageal carcinoma (ESCA) was drawn with Cytoscape software. The red rectangles and yellow triangles represented the miRNAs and TFs, respectively. The solid lines denoted the regulatory links among these factors.

**Figure 13:**
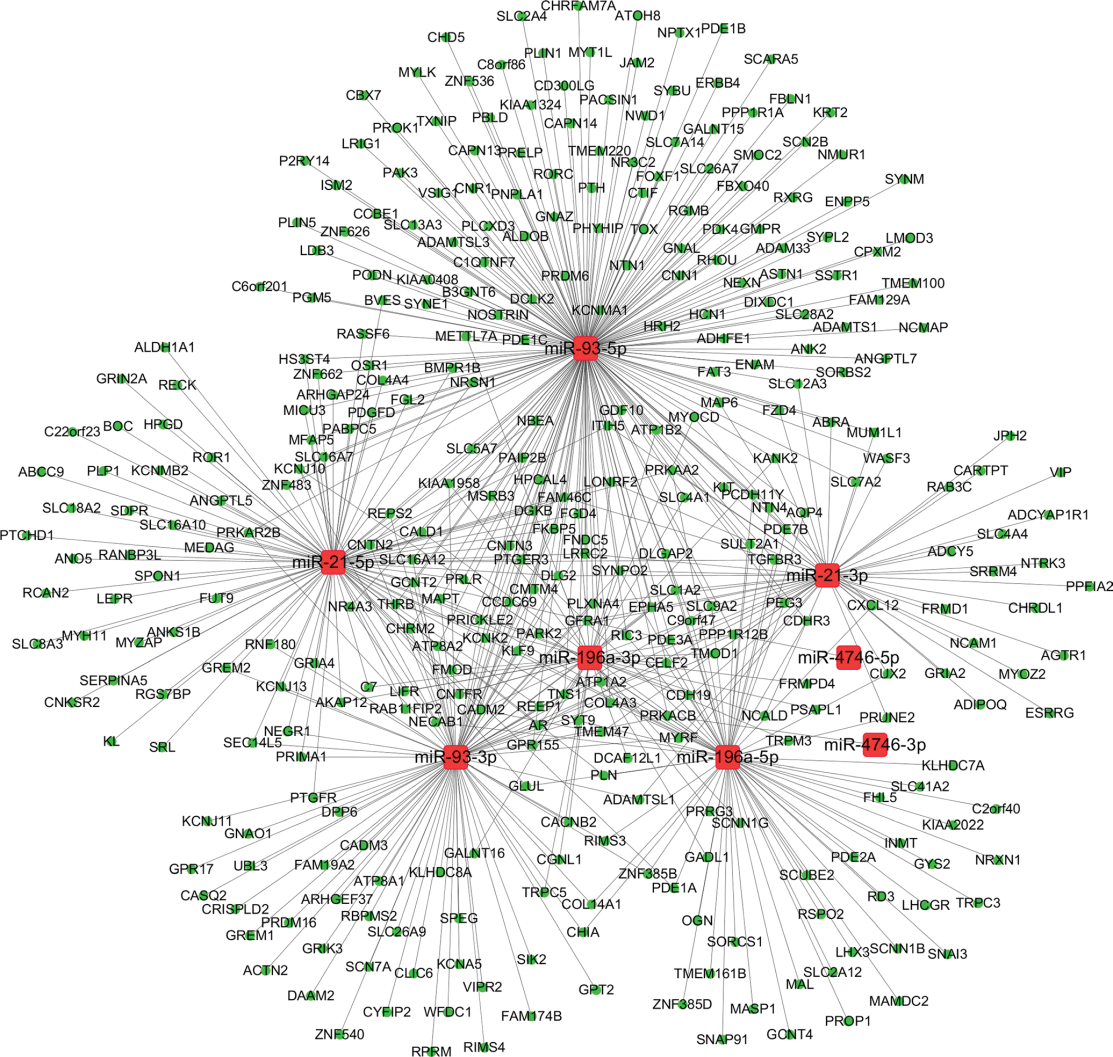
The regulatory network of miRNA-gene in esophageal carcinoma (ESCA) The regulatory network of miRNA-gene was analyzed using Cytoscape software. The round rectangles and ellipses represented the miRNAs and genes, respectively. The red and green colors represented the relatively high and low expression, respectively.

We predicted the target TFs of the four DEMs using CircuitsDB and TransmiR. The 38 pairs of the miRNA-TF network were constructed with the DEMs and 37 TFs (Table 2). The miRNA-TF and miRNA-gene regulatory networks were established with the 38 miRNA-TF pairs (Figure 12) and 560 miRNA-gene pairs (Figure 13) using Cytoscape software.

## DISCUSSION

It is well-known that TCGA, which has profiled a large number of malignancies at the DNA, RNA and protein levels, provides an excellent tool for cancer research. Considering the miRNA profile in ESCA, Zhan et al. [7] utilized the TCGA data from July 2014. The cohort only consisted of 70 cases of ESCA with mRNA data and 72 cases with miRNA data. They identified 2962 DEGs and 45 DEMs in ESCA. Subsequently, they performed gene function and signaling pathway analyses using GO and KEGG methods. They also established the TF-miRNA-gene network and observed that core promoter-binding protein (CPBP), nuclear factor of activated T-cells (NFAT-1), and miR-30c-5p, were located in the central hub of the network. However, Zhan et al. [7] did not explore the clinical significance of the DEMs, their prognostic value or their diagnostic values. Additionally, Zhao et al. [8]also screened for the presence of DEMs in ESCA based on the TCGA data from June 2015, consisting of 187 ESCA and 13 normal esophageal samples, which included 1046 miRNA expression values. They investigated the prognostic value of DEMs but not their diagnostic value. Thus, no study has explored the diagnostic value of DEMs in ESCA from TCGA data. Compared with previous studies by Zhan et al. [7] and Zhao et al. [8], the latest update of TCGA data with high throughput analysis of miRNAs was retrieved and assessed in the current study. Herein, 187 cases of ESCA and 13 cases of normal esophageal tissues with 1881 miRNAs detected with miRNA-seq were analyzed in the current study, which increased the reliability of our results. More importantly, we highlighted the diagnostic values of several core DEMs for ESCA, including miR-93, miR-21, miR-4746, miR-196a-1 and miR-196a-2.

Early diagnosis plays a critical role in the prevention and treatment of cancer, including ESCA. Using an ROC analysis, we found that the aforementioned five DEMs could be considered biomarkers for the diagnosis of ESCA. Importantly, the diagnostic values of these DEMs could be validated based on independent microarray data from GEO, as shown by rROC and SMD. More interestingly, combining these five miRNAs resulted in an extremely high diagnostic value with the AUC of 0.985. This miRNA-pool offers a great potential for the clinical detection and early screening of ESCA. In addition, we also attempted to assess the prognostic value of the top five DEMs in ESCA. Kaplan-Meier survival analysis was performed to estimate the prognostic values of the top five DEMs. Unfortunately, the top five DEMs did not gain any significant prognostic significance (Supplementary Figure 1).

Among these five DEMs, the one with the highest AUC was miR-93, which has been reported to be frequently dysregulated in various cancers, including gastric [9], liver [10], colorectal [11], breast [12], ovarian [13], cervical [14], prostate [15], bladder cancer [16] and bone sarcoma [17]. MiR-93 acts as a tumor promoter and may also be used as a potential biomarker for esophageal squamous cell carcinoma (ESCC) [18, 19], which was in complete agreement with the current finding based on TCGA data.

In addition, miR-21 has also been well studied in cancers, and several meta-analyses have reported its clinical value [20–29]. The up-regulation of miR-21 can predict an unfavorable prognosis in ESCA, as confirmed by three independent meta-analyses [30–32]. Several studies also found that the up-regulation of miRNA-21 could serve as a potential diagnostic biomarker [33–36], which is consistent with our result using TCGA data.

MiR-4746 was discovered by Persson et al. [37] using the first extensive next-generation sequencing analysis of miRNA expression in breast cancer. However, the clinical role of miR-4746 in cancers remains largely unknown. Hence, as a new member of the miRNA family, miR-4746 needs to be further studied, especially in terms of its clinical value in cancers, including ESCA. In our study, we identified two prospective target genes of PRKACB and PPP1R12B for miR-4746. PRKACB has been found to be a critical effector of the cAMP/PKA-related signal pathway and thus participate in several cell processes such as cell proliferation, differentiation, apoptosis, metabolism and gene transcription. Chen et al. [38] detected the expression of PRKACB in non-small lung cancer and adjacent non-cancer tissues using qRT-PCR, and found that PRKACB was down-regulated in cancerous tissues. The authors also demonstrated that PRKACB was a tumor suppressor gene through promoting tumor cells apoptosis and inhibiting tumor cells proliferation and invasion. Sigloch et al. [39] discovered that PRKACB was a direct target of miR-200c, and the down-regulated PRKACB could suppress the breast cancer cell migration. Zhou et al. [40] proposed that PPP1R12B was down-expressed in colorectal cancer tissues and might be a tumor suppressive gene. However, the molecular mechanisms of PRKACB and PPP1R12B in ESCA were still unknown, and the correlation between miR-4746 and PRKACB as well as PPP1R12B has either not been validated so far. In our investigation, miR-4746 was up-regulated, while PRKACB and PPP1R12B were down-regulated in ESCA and clear inverse correlations between miR-4746 and PRKACB, PPP1R12B could be noted (Figure 10E). We hypothesized that the overexpression of miR-4746 could repress the expression of PRKACB and PPP1R12B and further promote the occurrence and development of ESCA. Further and stricter experiments are warranted to confirm our speculation.

MiR-196 as a cell death-related microRNA was involved in the processes of apoptosis and autophagy [41]. MiR-196a binding-site SNP was reported to regulate RAP1A expression, which contributed to ESCA risk and metastasis [42]. A functional variation in pre-microRNA-196a has been correlated with the susceptibility of Chinese Han to ESCA risk [43]. MiR-196a has also been related to the development of Barrett's esophagus to esophageal adenocarcinoma [44, 45]. However, its expression level and clinical significance have not been reported in ESCA, which remains to be further investigated.

The circulating level of miRNAs has great clinical significance as non-invasive biomarkers for the early detection of ESCA. Some studies showed plasma/serum miR-21 was upregulated in ESCA patients than controls by using RT-PCR, and the area under the curve (AUC) were 0.812 [36], 0.690 [46] and 0.618 [47]. Moreover, upregulated miR-21 was detected in saliva supernatants with the AUC of 0.698 [48] and exosomes [49]. Not only miR-21, but also miR-23a [50], miR-506 [51], miR-718 [52] and miR-216a/b [53] were found as circulating biomarkers for diagnosis of ESCC. A meta-analysis [54] including 27 studies reported that circulating miRNAs with a combined AUC of 0.87 could be used as a biomarkers for early diagnosis of ESCC. MiRNAs presented in blood or other tissue fluid in a stable form [55]. Therefore, circulating miRNAs can be candidates for noninvasive diagnosis of ESCA. However, the circulating level of miR-93, miR-4746, miR-196a-1 and miR-196a-2 remains un-reported and needs to be detected in the future.

To further explore the possible molecular mechanisms of the five DEMs in the progression of ESCA, we performed PPI analysis, GO and KEGG pathway analysis of the overlapping genes. The top 10 hub genes (ADCY5, PRKACB, CXCL12, GPR17, NMUR1, AR, CNR1, CHRM2, GNAZ and PTGER3) were identified from the PPI network. Among these hub genes, CXCL12 expression has been shown to stimulate ESCC proliferation and associated with poor prognosis [56]. CXCL12 was also highly associated with ESCC development based upon graph-clustering and GO-term analysis [57]. Sato et al. reported that DNA methylation of ADCY5 was correlated with recurrence of lung adenocarcinoma [58]. Harten et al. found that Neuromedin U (NMU) combined with NMUR1 to stimulate migration of renal cancer cells [59]. Furthermore, we discovered that these overlapping genes were mainly involved in the plasma membrane, calcium signaling pathway and neuroactive ligand-receptor interaction, which gave us a direction to explore the molecular mechanisms of these DEMs in the tumorigenesis and deterioration of ESCA. To preliminarily validate the potential targets of the DEMs, we observed inverse relationships between DEMs and some of the hub genes, however, the exact validation needs to be performed with experiments *in vitro* in the future.

The development of ESCA is a complex biological process. We summarized the target TFs and constructed the regulatory network of miRNA-TF. TGF-β1 and EGFR, known TFs, may play significant roles in the progression of ESCA. TGF-β1 promotes the invasion and migration of sphere-forming stem-like cells in esophageal cancer [60]. TGF-β1 also induced the epithelial to mesenchymal transition (EMT) via the regulation of PTEN/PI3K signaling pathway in ESCC [61]. EGFR/AKT signaling pathway may play a crucial part in promoting ESCC development [62]. In addition, the expression of EGFR has been related to the prognosis of esophageal cancer [63–65]. Thus, DEMs may influence the TFs and then regulate the tumor development in ESCA. However, the function of miRNA-TF also requires further verification with *in vitro* and *in vivo* experiments.

## MATERIALS AND METHODS

### DEMs and DEGs of ESCA from TCGA data

The ESCA miRNA-Seq and RNA-Seq data were downloaded from the TCGA database using The GDC Data Portal (https://gdc-portal.nci.nih.gov/). The number of miRNA and mRNA expression values were 1881 and 60483. The miRNA expression data included a total of 200 samples consisting of 187 ESCA (90 esophageal adenocarcinoma (EAC) and 97 ESCC) and 13 normal esophageal samples. The mRNA expression data included a total of 173 samples consisting of 162 ESCA (80 EAC and 82 ESCC) and 11 normal esophageal samples. The sequencing data were all publicly available; therefore, no ethical issues were involved. The edgeR package in Bioconductor was used to screen the DEMs and DEGs in ESCA and normal esophageal tissue samples. The edgeR package is based on the negative binomial (NB) distribution, which can correct the overdispersion problem in RNA-seq data by using a Poisson model and a Bayes procedure [66, 67]. The data with expression values of zero were removed. The miRNAs and genes were deemed to be DEMs and DEGs if |log_2_FoldChange| > 1 [68] and |log_2_FoldChange| >1.5 [69], respectively, both with *p*-value < 0.05 and false discovery rate (FDR) < 0.05. Analysis of the diagnostic role of DEMs

We evaluated the differential expression level of DEMs using the scatter diagram software by GraphPad Prism (version 6.01) and employed *t*-test for statistical comparison. The ROC analysis of the DEMs was performed using MedCalc software [70]. The top five DEMs with the highest diagnostic performances were selected for further analysis. The sROC curves for the top five DEMs were generated using Meta-DISc software [71]. The AUC was calculated for the individual DEMs and their combination.

### Validation of the top five DEMs based on GEO miRNA microarray datasets

The ESCA miRNA microarray datasets were collected from GEO (http://www.ncbi.nlm.nih.gov/geo/) on the basis of the search terms: (esophageal OR esophagus OR esophag* OR ESCC OR ESCA OR EC OR EA) AND (cancer OR carcinoma OR tumo* OR neoplas* OR malignan* OR adenocarcinoma) AND (miR OR miRNA OR microRNA OR “non coding RNA” OR “non-coding RNA”). The search date was up to February 15, 2017.

We selected available datasets in accordance with the following inclusion criteria: (1) the samples in each datasets must be from human beings; (2) the patients must be diagnosed as esophageal carcinoma pathologically; (3) the records must provide miRNA expression data for both cancerous and non-cancerous specimens; (4) both the cancer and non-cancer groups must include at least three samples, respectively [72].

Two reviewers (Jiang-hui Zeng and Dan-dan Xiong) independently extracted detailed information from included datasets, with any divergence was confirmed by conversation with a third and fourth researchers (Gang Chen and Dian-zhong Luo). The following characteristics were collected: data source, platform, first author, publication years, region, cancer type, sample source, number of patients for both cancer and normal groups and expression values of the top five DEMs.

The mean and standard deviation of the top five DEMs expression values in cancer and non-cancer groups were calculated using SPSS 20.0 (IBM, New York, USA). The pooled standard mean deviation (SMD) and its corresponding 95% confidence interval (95% CI) were further evaluated using STATA, version 12.0 (StataCorp, College Station, TX, USA). The SMD > 0 demonstrated that the DEMs were highly expressed in cancer samples. The *P* < 0.05 (two-sided) was considered statistically significant.

### Collection of potential target genes for DEMs

We predicted the potential target genes of the top five DEMs using miRWalk 2.0 [73], which included 12 databases (miRWalk [74], Microt4 [75], miRanda [76], mirbridge [77], miRDB [78], miRMap [79], miRNAMap [80], Pictar2 [81], PITA [82], RNA22 [83], RNAhybrid [84] and TargetScan [85]). The genes predicted by six or more databases were regarded as potential target genes for each DEM. We also combined all predicted genes together as a group, which could reflect the known potential targets in ESCA in general. Then, the intersection between the above predicted genes and the DEGs of TCGA was determined using Venn diagram online tool. For those four up-regulated DEMs (miR-93, miR-21, miR-4746, miR-196a), we selected the down-regulated DEGs for the intersection due to the negative relationship between the miRNA and its target gene based upon sequence complementation.

### PPI network construction

PPI network of the overlapping DEGs was established using the Search Tool for the Retrieval of Interacting Genes (STRING) database (http://www.string-db.org/). The interactions procured included known interactions and predicted interactions.

### Validation of the top five DEMs target genes based on TCGA datasets

PPI network was performed to the overlapping genes of each DEM (miR-93, miR-21, miR-196a-1 and miR-196a-2). Pearson's correlation between the top five hub genes and each DEM was performed by R language.

### Functional annotation

The Database for Annotation Visualization and Integrated Discovery (DAVID) online tool (https://david.ncifcrf.gov/) was used to conduct the functional and pathway enrichment analyses in our study. We performed GO and KEGG pathway enrichment analyses to detect the potential biological functions and pathways of the overlapping genes of the four DEMs in ESCA.

### Identification of miRNA-transcription factors (TFs)

CircuitsDB is a web-server established to search human and mouse mixed miRNA/TF Feed-Forward regulatory circuits [86]. The transcription factor-miRNA regulation database (TransmiR) provides a valuable resource for the study of TF-miRNA regulation [87]. We thus obtained miRNA-TF regulatory relations using CircuitsDB and TransmiR.

### Networks of miRNA-TF and miRNA-gene

Based on the miRNA-TF pairs and miRNA-gene pairs, we constructed the regulatory networks of miRNA-TF and miRNA-gene in ESCA, which we visualized using Cytoscape (v3.4.0) software.

## CONCLUSIONS

In the current study, we screened for the presence of DEMs and DEGs in normal esophageal and ESCA samples in the genome-wide miRNA expression profiles from TCGA and GEO. We selected the top five miRNAs with the highest AUC value to construct the networks of miRNA-TF and miRNA-Gene. Our study may provide a meaningful contribution to exploring the role of novel DEMs in ESCA pathogenesis and improving the early diagnosis of ESCA.

## SUPPLEMENTARY MATERIALS FIGURE AND TABLES




