# Supplementary material for: Identification of molecular targets for esophageal carcinoma diagnosis using miRNA-seq and RNA-seq data from The Cancer Genome Atlas: a study of 187 cases
Source: Oncotarget. 2017 Mar 9;8(22):35681–99. doi: 10.18632/oncotarget.16051 (PMC5482608; doi:10.18632/oncotarget.16051)
Supplement: Supplementary file 1 [file oncotarget-08-35681-s001.pdf]

Identification of molecular targets for esophageal carcinoma diagnosis using miRNA-seq and RNA-seq data from The Cancer Genome Atlas: a study of 187 cases

Supplementary Materials

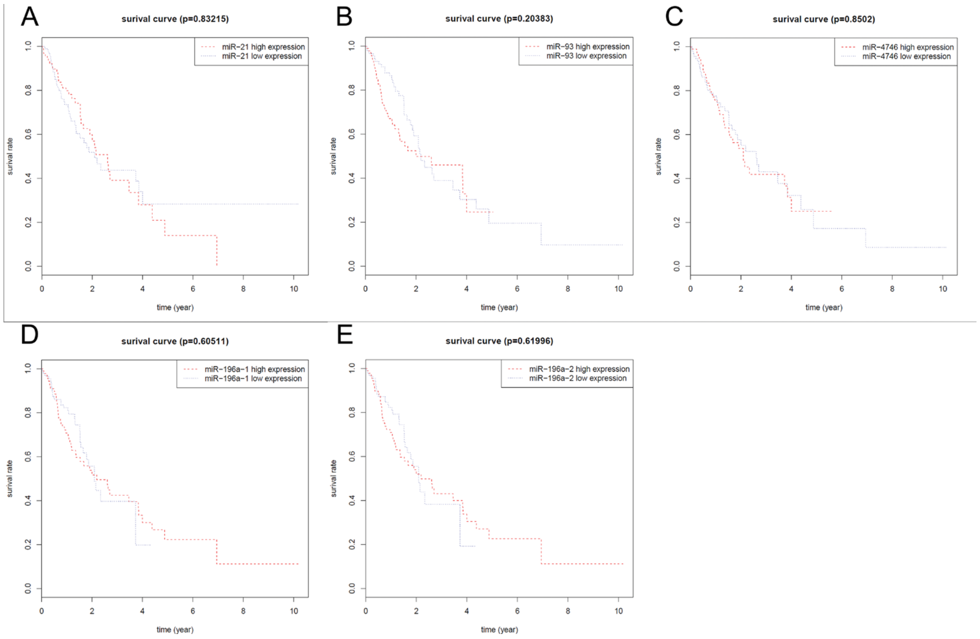

Supplementary Figure 1: Kaplan-Meier survival analysis of the top five differentially expressed miRNAs (DEMs). Kaplan-Meier survival analysis plots were drawn using R language. (**A**) miR-21, (**B**) miR-93, (**C**) miR-4746, (**D**) miR-196a-1, (**E**) miR-196a-2.

Supplementary Table 1: 136 differentially expressed miRNAs (DEMs) in esophageal carcinoma (ESCA). See Supplementary_Table_1

Supplementary Table 2: Identification and prediction of overlapping genes from differentially expressed genes (DEGs)

miRNA ID	Overlapping Genes
**miR-93-3p**	SEC14L5, ZNF385B, PRDM16, CNTN2, KCNJ13, GNAO1, NEGR1, REPS2, SLC9A2, HPCAL4, GCNT2, FKBP5, SIK2, PRKACB, NR4A3, PARK2, KLF9, REEP1, DPP6, DAAM2, PLXNA4, KLHDC8A, SPEG, GREM2, CGNL1, TRPC5, FAM19A2, PRIMA1, CMTM4, GRIK3, CACNB2, SCN7A, GPR17, CCDC69, GFRA1, FAM46C, ATP8A2, RIMS4, GRIA4, WFDC1, KCNJ11, RPRM, CYFIP2, ADAMTSL1, FAM174B, PTGER3, SYNPO2, KIAA1958, SLC16A12, RNF180, EPHA5, COL4A3, MAPT, ZNF540, PTGFR, TMEM47, RIMS3, SLC1A2, TNS1, KCNJ10, RBPMS2, GREM1, ACTN2, PRICKLE2, VIPR2, CNTN3, PRKAR2B, ARHGEF37, DLG2, PPP1R12B, ATP1A2, MYRF, CADM3, UBL3, FNDC5, COL14A1, ATP8A1, SLC26A9, CHIA, KCNA5, CHRM2, PAIP2B, TMOD1, GPT2, CASQ2, CRISPLD2, SYT9, AR, GALNT16, CLIC6
**miR-93-5p**	ABRA, KCNMA1, LDB3, CPXM2, MSRB3, PDE1C, NOSTRIN, PHYHIP, PODN, PABPC5, GMPR, GDF10, KIAA1324, THRB, CNTN2, C8orf86, LONRF2, PBLD, SORBS2, RIC3, HRH2, NTN4, SLC9A2, HPCAL4, FKBP5, ZNF536, MAP6, NRSN1, NR4A3, PARK2, PLXNA4, SMOC2, KIAA0408, SLC12A3, SLC5A7, ADHFE1, NBEA, MYT1L, CHRFAM7A, SYNE1, CMTM4, PTH, SYBU, PEG3, B3GNT6, CCBE1, PGM5, ASTN1, FAM46C, CALD1, ARHGAP24, SLC13A3, PDE1B, FZD4, SLC26A7, SYNM, PTGER3, ERBB4, ITIH5, SLC16A7, CTIF, CAPN14, FGD4, EPHA5, AQP4, SLC7A14, COL4A3, CNR1, ANK2, SLC1A2, PAK3, DGKB, ATP1B2, TNS1, KCNJ10, WASF3, TXNIP, PRICKLE2, TMEM100, SLC7A2, CNTN3, PRKAA2, PRKAR2B, CAPN13, PCDH11Y, ADAM33, COL4A4, ENPP5, CXCL12, NTN1, JAM2, ISM2, SLC2A4, ZNF626, CHD5, ZNF662, SCN2B, HS3ST4, ADAMTSL3, MUM1L1, SLC4A1, GNAL, NWD1, HCN1, CNN1, LMOD3, ATOH8, MFAP5, DIXDC1, LRRC2, VSIG1, PDGFD, BVES, KIT, DCLK2, C1QTNF7, REPS2, PRDM6, TOX, GCNT2, PLCXD3, FMOD, LRIG1, PRKACB, PDE3A, KLF9, PDK4, MYOCD, PDE7B, P2RY14, GALNT15, GPR155, NR3C2, RHOU, GNAZ, MYLK, CBX7, ZNF483, CELF2, PLIN5, KANK2, CCDC69, TGFBR3, PPP1R1A, RORC, GFRA1, ATP8A2, PRELP, FGL2, MICU3, RGMB, SYNPO2, FAM129A, KIAA1958, SLC16A12, CADM2, ANGPTL7, SYPL2, ENAM, MAPT, FBXO40, FBLN1, SULT2A1, PNPLA1, CD300LG, CDHR3, PLIN1, KRT2, RXRG, NMUR1, KCNK2, DLG2, PPP1R12B, SCARA5, ATP1A2, SLC28A2, PROK1, ADAMTS1, PACSIN1, OSR1, FNDC5, NEXN, FOXF1, PRLR, FAT3, METTL7A, CHRM2, SSTR1, PAIP2B, TMEM220, C6orf201, NCMAP, NPTX1, DLGAP2, BMPR1B, ALDOB, SYT9, RASSF6, C9orf47
**miR-21-3p**	ABRA, MUM1L1, SRRM4, NCAM1, LONRF2, LRRC2, JPH2, MYOZ2, KIT, SLC9A2, PRKACB, PDE3A, MAP6, PARK2, ADCYAP1R1, REEP1, MYOCD, PLXNA4, SLC12A3, PPFIA2, VIP, PEG3, KANK2, TGFBR3, GFRA1, FAM46C, FZD4, SLC4A4, ADAMTSL1, CHRDL1, ITIH5, SYNPO2, RAB3C, ADCY5, ESRRG, TMEM47, SLC1A2, AGTR1, PRUNE2, NTRK3, WASF3, CUX2, SLC7A2, PRKAA2, DLG2, PPP1R12B, MYRF, FRMD1, CXCL12, CARTPT, TMOD1, NCALD, AR, C9orf47, GRIA2, ADIPOQ
**miR-21-5p**	ANGPTL5, SEC14L5, ANO5, PABPC5, KL, THRB, CNTFR, MFAP5, CNTN2, LONRF2, KCNJ13, LRRC2, PDGFD, BVES, NEGR1, REPS2, GCNT2, FKBP5, PDE3A, NRSN1, GRIN2A, C22orf23, KLF9, CNKSR2, REEP1, SRL, ABCC9, GREM2, SLC5A7, NBEA, PRIMA1, AKAP12, ZNF483, MYZAP, TGFBR3, CDH19, BOC, FAM46C, CALD1, ARHGAP24, GRIA4, PLP1, PTCHD1, RGS7BP, ROR1, SERPINA5, MEDAG, PTGER3, FGL2, MICU3, ITIH5, PLN, SLC16A7, SLC8A3, CADM2, RNF180, FGD4, RAB11FIP2, RCAN2, PTGFR, LEPR, ANKS1B, SPON1, MYH11, DGKB, TNS1, KCNJ10, HPGD, C7, PDE1A, PRICKLE2, CNTN3, PRKAA2, KCNK2, DLG2, RANBP3L, SDPR, COL4A4, LIFR, FUT9, OSR1, SLC18A2, GLUL, NECAB1, RECK, ALDH1A1, PRLR, PAIP2B, KCNMB2, BMPR1B, ZNF662, HS3ST4, AR, RASSF6, SLC16A10
**miR-4746-3p**	PRKACB
**miR-4746-5p**	PPP1R12B
**miR-196a-3p**	SLC4A1, MSRB3, GADL1, THRB, CNTFR, LONRF2, FRMPD4, FMOD, MYOCD, GPR155, CGNL1, TRPC5, AKAP12, CACNB2, CELF2, TRPM3, KANK2, PRRG3, SCNN1G, PSAPL1, SYNPO2, KIAA1958, CADM2, FGD4, RAB11FIP2, MAPT, SLC1A2, ATP1B2, C7, DLG2, PPP1R12B, LIFR, METTL7A, TMOD1, NCALD, DCAF12L1, AR, GALNT16, C9orf47
**miR -196-5p**	PROP1, ZNF385B, RSPO2, RIC3, NTN4, SLC9A2, SCNN1B, PDE3A, FHL5, NRXN1, KLF9, PDE7B, KLHDC7A, OGN, GYS2, CELF2, PEG3, TRPM3, TGFBR3, CDH19, MASP1, CALD1, C2orf40, PTGER3, ZNF385D, SYNPO2, KIAA2022, PLN, CADM2, TMEM161B, MAL, AQP4, RD3, COL4A3, RIMS3, SLC1A2, SULT2A1, CDHR3, KCNJ10, PRUNE2, CUX2, PDE1A, SNAI3, PRICKLE2, PRKAA2, KCNK2, SORCS1, PPP1R12B, PDE2A, LHCGR, ATP1A2, PCDH11Y, SLC2A12, LHX3, CXCL12, GLUL, NECAB1, COL14A1, SLC41A2, PRLR, CHIA, INMT, GCNT4, SCUBE2, DLGAP2, MAMDC2, SYT9, SNAP91, TRPC3, C9orf47
