# Supplementary material for: Identification of molecular targets for esophageal carcinoma diagnosis using miRNA-seq and RNA-seq data from The Cancer Genome Atlas: a study of 187 cases
Source: Oncotarget. 2017 Mar 9;8(22):35681–99. doi: 10.18632/oncotarget.16051 (PMC5482608; doi:10.18632/oncotarget.16051)
Supplement: Supplementary file 2 [file oncotarget-08-35681-s002.docx]

**Supplementary Table 1. 136 differentially expressed miRNAs (DEMs) in esophageal carcinoma (ESCA)**

| miRNA ID | LogFC* | *p*-value | FDR** | AUC*** |
| --- | --- | --- | --- | --- |
| miR-93 | 1.394654953 | 4.44E-08 | 3.18E-06 | 0.953 |
| miR-21 | 1.246193842 | 2.81E-08 | 2.12E-06 | 0.928 |
| miR-4746 | 1.896011225 | 1.72E-07 | 9.94E-06 | 0.915 |
| miR-196a-1 | 1.993005619 | 0.000557766 | 0.007777738 | 0.906 |
| miR-196a-2 | 1.943227278 | 0.000604741 | 0.008131613 | 0.906 |
| miR-1468 | -1.772235407 | 1.11E-07 | 6.97E-06 | 0.904 |
| miR-301b | 2.241901263 | 3.53E-06 | 0.000136312 | 0.899 |
| miR-877 | 1.754576071 | 8.74E-06 | 0.000299107 | 0.898 |
| miR-135a-2 | -2.872963869 | 7.46E-11 | 1.02E-08 | 0.892 |
| miR-106b | 1.240499712 | 8.15E-07 | 3.81E-05 | 0.89 |
| miR-4664 | 4.028887486 | 4.16E-07 | 2.16E-05 | 0.889 |
| miR-301a | 1.182941942 | 2.02E-05 | 0.000563583 | 0.888 |
| miR-17 | 1.033326155 | 4.77E-06 | 0.000175258 | 0.884 |
| miR-135a-1 | -2.736882064 | 1.21E-09 | 1.40E-07 | 0.882 |
| miR-18a | 1.619024802 | 1.13E-05 | 0.00036778 | 0.881 |
| miR-335 | 1.689839612 | 5.27E-06 | 0.000188867 | 0.873 |
| miR-204 | -3.698324817 | 5.36E-17 | 2.69E-14 | 0.872 |
| miR-4652 | 3.938365244 | 1.78E-07 | 9.94E-06 | 0.872 |
| miR-421 | 1.341497303 | 1.05E-05 | 0.000350283 | 0.87 |
| miR-615 | 2.134931947 | 4.06E-05 | 0.001001998 | 0.87 |
| miR-937 | 2.391401812 | 1.23E-05 | 0.000379284 | 0.869 |
| miR-1304 | 2.478355915 | 1.49E-05 | 0.000432103 | 0.866 |
| miR-378c | -1.497114047 | 1.22E-08 | 9.65E-07 | 0.863 |
| miR-942 | 1.138137566 | 3.79E-05 | 0.000952212 | 0.863 |
| miR-135b | 2.216470642 | 1.15E-05 | 0.00036778 | 0.856 |
| miR-455 | 1.328448605 | 5.51E-05 | 0.001257181 | 0.854 |
| miR-196b | 1.899610469 | 0.000377144 | 0.005795697 | 0.853 |
| miR-30e | -1.218537007 | 1.83E-12 | 4.60E-10 | 0.852 |
| miR-139 | -2.034271233 | 3.05E-17 | 2.30E-14 | 0.847 |
| miR-550a-1 | 1.504389105 | 9.90E-05 | 0.002071763 | 0.846 |
| miR-345 | 1.103778261 | 4.33E-05 | 0.001051959 | 0.845 |
| miR-1301 | 1.04836907 | 0.000240664 | 0.004091732 | 0.84 |
| miR-940 | 1.509942205 | 0.000119099 | 0.002423829 | 0.84 |
| miR-29c | -1.761926149 | 1.16E-08 | 9.65E-07 | 0.838 |
| miR-30a | -1.776128401 | 3.85E-11 | 5.81E-09 | 0.837 |
| miR-181b-1 | 1.20343134 | 3.69E-05 | 0.000942568 | 0.837 |
| miR-129-1 | -1.876791424 | 0.000127739 | 0.002564995 | 0.836 |
| miR-383 | -2.365307631 | 7.67E-05 | 0.001674128 | 0.834 |
| miR-1180 | 1.240833317 | 0.000155191 | 0.002958451 | 0.833 |
| miR-3682 | 1.267871428 | 0.00050449 | 0.007100584 | 0.829 |
| miR-146b | 1.049360663 | 0.000262802 | 0.004397557 | 0.827 |
| miR-224 | 3.114004206 | 4.66E-06 | 0.000175258 | 0.827 |
| miR-550a-3 | 1.485216154 | 0.000426473 | 0.006359085 | 0.822 |
| miR-181b-2 | 1.139801962 | 8.80E-05 | 0.001866281 | 0.821 |
| miR-483 | 4.107223045 | 0.000157646 | 0.002967678 | 0.817 |
| miR-664a | -1.106129764 | 7.50E-09 | 6.65E-07 | 0.814 |
| miR-3652 | 2.292986141 | 0.000890148 | 0.01107825 | 0.811 |
| miR-550a-2 | 1.133988368 | 0.00196548 | 0.020555648 | 0.81 |
| miR-452 | 1.981914413 | 0.000276453 | 0.004575148 | 0.808 |
| miR-30c-1 | -1.622556733 | 1.38E-15 | 4.16E-13 | 0.807 |
| miR-3662 | 3.471219717 | 0.000295524 | 0.004820629 | 0.807 |
| miR-3677 | 1.270608778 | 0.000297688 | 0.004820629 | 0.804 |
| miR-133a-1 | -2.978898023 | 4.86E-12 | 1.05E-09 | 0.802 |
| miR-378d-1 | -1.540181578 | 3.13E-06 | 0.00012742 | 0.802 |
| miR-375 | -2.38202974 | 7.77E-06 | 0.00027222 | 0.801 |
| miR-4661 | 1.370750763 | 0.00205933 | 0.021388624 | 0.795 |
| miR-133a-2 | -2.955119128 | 8.57E-12 | 1.61E-09 | 0.794 |
| miR-6854 | 1.320000178 | 0.002799041 | 0.027195846 | 0.792 |
| miR-767 | 7.583176722 | 1.23E-06 | 5.16E-05 | 0.791 |
| miR-129-2 | -1.672539788 | 0.000241809 | 0.004091732 | 0.79 |
| miR-636 | 2.217070079 | 0.00045094 | 0.006467775 | 0.787 |
| miR-210 | 1.563532119 | 0.000483308 | 0.006866622 | 0.786 |
| miR-6891 | 3.755537465 | 5.38E-05 | 0.001245845 | 0.783 |
| miR-30c-2 | -1.299689291 | 2.13E-09 | 2.01E-07 | 0.781 |
| miR-490 | -4.188762519 | 1.17E-15 | 4.16E-13 | 0.78 |
| miR-3690-1 | 1.756209279 | 0.001392362 | 0.016006853 | 0.78 |
| miR-183 | 1.234499868 | 0.001913942 | 0.020156618 | 0.778 |
| miR-548f-1 | 4.267584678 | 0.000436286 | 0.006416185 | 0.775 |
| miR-105-2 | 6.642786337 | 1.44E-05 | 0.000424728 | 0.774 |
| miR-1228 | 1.276647439 | 0.002871314 | 0.02759272 | 0.774 |
| miR-34c | 2.162366835 | 0.00243821 | 0.02469092 | 0.771 |
| miR-1-1 | -2.237325943 | 5.89E-07 | 2.96E-05 | 0.77 |
| miR-3150b | 2.170808546 | 0.002501276 | 0.024986698 | 0.768 |
| miR-573 | 2.928645201 | 0.001087049 | 0.013096771 | 0.768 |
| miR-148a | -2.621344728 | 4.15E-31 | 6.25E-28 | 0.765 |
| miR-584 | 1.391504268 | 0.001298727 | 0.01528033 | 0.765 |
| miR-1-2 | -2.311943498 | 1.54E-07 | 9.29E-06 | 0.763 |
| miR-1258 | -1.773069246 | 6.13E-05 | 0.001358081 | 0.762 |
| miR-6728 | 3.181999341 | 0.000228344 | 0.004045719 | 0.762 |
| miR-4521 | -1.291850518 | 0.00244286 | 0.02469092 | 0.761 |
| miR-1293 | 2.698641941 | 0.001755583 | 0.018956245 | 0.761 |
| miR-3152 | -1.864625276 | 0.004574468 | 0.039416533 | 0.76 |
| miR-3691 | 1.783922742 | 0.002198343 | 0.022521804 | 0.756 |
| miR-153-1 | -2.129496076 | 9.63E-08 | 6.31E-06 | 0.753 |
| miR-202 | -2.42709681 | 3.49E-06 | 0.000136312 | 0.753 |
| miR-3651 | 1.580170841 | 0.00581939 | 0.047890714 | 0.753 |
| miR-548d-1 | 3.117281591 | 0.000445378 | 0.006449414 | 0.749 |
| miR-378f | -1.545449727 | 0.000879636 | 0.011039427 | 0.747 |
| miR-675 | 2.750527839 | 0.001686997 | 0.018544655 | 0.747 |
| miR-4449 | 3.571863823 | 0.000875528 | 0.011039427 | 0.746 |
| miR-548d-2 | 3.148050638 | 0.000563019 | 0.007778956 | 0.746 |
| miR-4745 | 2.737618109 | 0.000754398 | 0.009794173 | 0.742 |
| miR-4517 | 2.28301015 | 0.003004844 | 0.028513497 | 0.739 |
| miR-5703 | 2.972704709 | 0.000573337 | 0.007849505 | 0.738 |
| miR-133b | -2.127944762 | 2.90E-05 | 0.000779334 | 0.736 |
| miR-3654 | 3.087525426 | 0.001495163 | 0.016930195 | 0.727 |
| miR-145 | -2.034775122 | 2.00E-09 | 2.01E-07 | 0.724 |
| miR-195 | -1.096471275 | 3.20E-05 | 0.000844477 | 0.723 |
| miR-6781 | 2.08434868 | 0.003010389 | 0.028513497 | 0.722 |
| miR-6874 | -1.426078613 | 0.000240738 | 0.004091732 | 0.719 |
| miR-676 | -1.165151155 | 0.003872056 | 0.035276515 | 0.715 |
| miR-125a | -1.116450753 | 8.57E-08 | 5.87E-06 | 0.713 |
| miR-1910 | 2.910821265 | 0.001710755 | 0.018669546 | 0.712 |
| miR-5090 | 2.609704165 | 0.003388547 | 0.03130768 | 0.706 |
| miR-105-1 | 6.110207015 | 2.52E-05 | 0.00069044 | 0.705 |
| miR-7-3 | 1.840468327 | 0.004599676 | 0.039416533 | 0.705 |
| miR-1269a | 3.977385527 | 0.002876532 | 0.02759272 | 0.703 |
| miR-612 | 3.432153894 | 0.003034519 | 0.028562408 | 0.701 |
| miR-6788 | -1.845366669 | 5.08E-05 | 0.001194312 | 0.697 |
| miR-378d-2 | -1.361239055 | 3.48E-05 | 0.000903871 | 0.695 |
| miR-6715b | 4.157210051 | 0.001891844 | 0.020064201 | 0.693 |
| miR-153-2 | -2.141822598 | 7.75E-10 | 9.73E-08 | 0.691 |
| miR-28 | -1.278835523 | 2.84E-11 | 4.75E-09 | 0.686 |
| miR-372 | 11.24850438 | 0.001037003 | 0.012594568 | 0.674 |
| miR-1225 | -1.809502623 | 0.000197427 | 0.003539585 | 0.671 |
| miR-4757 | -1.614531546 | 0.001232377 | 0.014729841 | 0.662 |
| miR-4770 | -1.850182085 | 0.000147029 | 0.002838783 | 0.657 |
| miR-512-1 | 5.87904805 | 0.004451942 | 0.038755056 | 0.654 |
| miR-5680 | -2.593812006 | 1.51E-09 | 1.63E-07 | 0.653 |
| miR-526b | 5.360068449 | 0.005677395 | 0.047238438 | 0.65 |
| miR-548ba | -2.025831546 | 0.000417018 | 0.006280291 | 0.649 |
| miR-516a-1 | 5.431815448 | 0.004606447 | 0.039416533 | 0.648 |
| miR-365a | -1.185219985 | 1.01E-06 | 4.36E-05 | 0.641 |
| miR-365b | -1.188422326 | 8.95E-07 | 3.96E-05 | 0.64 |
| miR-4510 | -2.276832204 | 3.62E-07 | 1.95E-05 | 0.636 |
| miR-6129 | -1.457225074 | 0.005452906 | 0.045622646 | 0.636 |
| miR-642a | -1.239292874 | 7.84E-05 | 0.001687224 | 0.632 |
| miR-3622a | -1.528764841 | 0.001762201 | 0.018956245 | 0.625 |
| miR-218-1 | -1.204323224 | 0.00018086 | 0.003321656 | 0.611 |
| miR-1265 | -2.33298478 | 0.000138969 | 0.002718015 | 0.607 |
| miR-218-2 | -1.180509341 | 0.000321543 | 0.005151527 | 0.604 |
| miR-100 | -1.225795201 | 0.000767089 | 0.009873815 | 0.592 |
| miR-143 | -1.430752055 | 1.25E-05 | 0.000379284 | 0.58 |
| miR-6507 | -2.00829705 | 0.00016887 | 0.00313974 | 0.564 |
| let-7c | -1.140505473 | 0.001608145 | 0.018073631 | 0.558 |
| miR-508 | 2.965397204 | 0.002782416 | 0.027195846 | 0.555 |

*FC: fold change **FDR: fault detection rate ***AUC: area under the ROC curve
